# What behaviour change techniques have been used to improve adherence to evidence-based low back pain imaging?

**DOI:** 10.1186/s13012-021-01136-w

**Published:** 2021-07-02

**Authors:** Amanda Hall, Helen Richmond, Andrea Pike, Rebecca Lawrence, Holly Etchegary, Michelle Swab, Jacqueline Y. Thompson, Charlotte Albury, Jill Hayden, Andrea M. Patey, James Matthews

**Affiliations:** 1 Primary Healthcare Research Unit, Faculty of Medicine, Memorial University, 300 Prince Phillip Drive, St. John’s, Newfoundland A1B 3V6 Canada; 2 Public Health, Institute of Applied Health Research, College of Medicine and Dentistry, University of Birmingham, Birmingham, B15 2TT UK; 3 Nuffield Department of Primary Care Health Sciences, University of Oxford, Oxford, England; 4 Department of Community Health and Epidemiology, Dalhousie University, Halifax, Nova Scotia Canada; 5 Centre for Implementation Research, Ottawa Hospital Research Institute, Ottawa, Canada; 6 School of Public Health, Physiotherapy & Sports Science, University College Dublin, Belfield, Dublin 4, Ireland

**Keywords:** Low back pain, Imaging, Evidence-based, Behaviour change techniques, Theoretical domains framework

## Abstract

**Background:**

Despite international guideline recommendations, low back pain (LBP) imaging rates have been increasing over the last 20 years. Previous systematic reviews report limited effectiveness of implementation interventions aimed at reducing unnecessary LBP imaging. No previous reviews have analysed these implementation interventions to ascertain what behaviour change techniques (BCTs) have been used in this field. Understanding what techniques have been implemented in this field is an essential first step before exploring intervention effectiveness.

**Methods:**

We searched EMBASE, Ovid (Medline), CINAHL and Cochrane CENTRAL from inception to February 1, 2021, as well as and hand-searched 6 relevant systematic reviews and conducted citation tracking of included studies. Two authors independently screened titles, abstracts, and full texts for eligibility and extracted data on study and intervention characteristics. Study interventions were qualitatively analysed by three coders to identify BCTs, which were mapped to mechanisms of action from the theoretical domains framework (TDF) using the Theory and Techniques Tool.

**Results:**

We identified 36 eligible studies from 1984 citations in our electronic search and a further 2 studies from hand-searching resulting in 38 studies that targeted physician behaviour to reduce unnecessary LBP imaging. The studies were conducted in 6 countries in primary (*n* = 31) or emergency care (*n* = 7) settings. Thirty-four studies were included in our BCT synthesis which found the most frequently used BCTs were ‘4.1 instruction on how to perform the behaviour’ (e.g. Active/passive guideline dissemination and/or educational seminars/workshops), followed by ‘9.1 credible source’, ‘2.2 feedback on behaviour’ (e.g. electronic feedback reports on physicians’ image ordering) and 7.1 prompts and cues (electronic decision support or hard-copy posters/booklets for the office). This review highlighted that the majority of studies used education and/or feedback on behaviour to target the domains of knowledge and in some cases also skills and beliefs about capabilities to bring about a change in LBP imaging behaviour. Additionally, we found there to be a growing use of electronic or hard copy reminders to target the domains of memory and environmental context and resources.

**Conclusions:**

This is the first study to identify what BCTs have been used to target a reduction in physician image ordering behaviour. The majority of included studies lacked the use of theory to inform their intervention design and failed to target known physician-reported barriers to following LBP imaging guidelines.

**Protocol Registation:**

PROSPERO CRD42017072518

**Supplementary Information:**

The online version contains supplementary material available at 10.1186/s13012-021-01136-w.

Contributions to the literature

- This is the first study to identify what behaviour change techniques have been used to reduce physician low back pain image ordering.
- We identified the majority of interventions used education based on imaging guidelines to target physician knowledge and skills in the hope of reducing low back pain image ordering.
- Our results highlight a need to utilise theory when designing interventions to ensure known barriers to low back pain imaging ordering behaviour are targeted.
- Our qualitative synthesis may help future researchers to better explore the effectiveness of single/combined behaviour change techniques for reducing physician low back pain image ordering.

## Introduction

Despite international guidelines and campaigns calling to reduce diagnostic imaging in low back pain (LBP) [1–3], recent evidence suggests that imaging has increased over the last 20 years [4]. Liberal use of LBP imaging results in avoidable costs to both healthcare systems and individual patients [5]. In addition to subjecting patients to unnecessary exposure to radiation, systematic reviews have shown that patients who received imaging without a clear clinical indication had longer recovery times, reported lower quality of life, and were more delayed in their return to work than those who were not imaged [6]. This has led to an increasing number of studies evaluating interventions that aim to change physician behaviour to reduce unnecessary imaging in LBP. For example, four relevant systematic reviews have evaluated interventions to change physician image-ordering behaviour for patients with LBP [7–10] (most of which included the same studies). Due to the heterogeneity of the interventions, none of these reviews included a meta-analysis. Narrative synthesis of the interventions, however, indicated variable effectiveness and all reviews concluded that there was insufficient evidence to identify which interventions were effective for reducing imaging rates for LBP.

While these interventions have attempted to change physician behaviour, most have not based their interventions on behaviour change theory. At least part of the reason why we see this variable effectiveness is likely due to their lack of theoretical underpinnings. Behaviour change theories such as the theoretical domains framework (TDF) can be used to identify barriers and help select intervention strategies (or behaviour change techniques (BCTs) to address those barriers. Indeed, a host of national health and research organisations including the National Institutes for Clinical Excellence (NICE) and the Medical Research Framework (MRC) [11, 12] suggest that behaviour change interventions should be rooted in theory—specifically, in a theoretical model of behaviour change. By using a theoretical framework, we can improve our understanding of the behaviour we want to change, understand the mechanism by which the behaviour can be changed and, thus, choose BCTs that are most suitable for the target behaviour and context [13].

In this context, understanding why physicians fail to change their ordering practices may provide helpful insights into how we can best intervene to support that change. To this end, our team conducted a systematic review synthesising 11 qualitative studies that asked physicians about the factors that might influence their ordering behaviour. We used the TDF to guide our analysis. Originally developed by Michie et al. to identify factors influencing health professional’s practice behaviour, the TDF is a synthesis of over 128 key theoretical constructs from 36 behaviour change theories into overarching domains presented in a single framework [14–17]. Depending on the behaviour in question and contextual factors, some domains are likely to be more important than others. Across all studies, conducted worldwide, we found high-level evidence that 3 TDF domains influenced LBP imaging behaviour: (i) Social influence (patient demand), (ii) Beliefs about consequences (lack of ability to explain why an image is not needed) and (iii) Environmental context and resources (lack of time) [18].

While previous reviews have attempted to examine the effectiveness of the interventions to reduce inappropriate imaging, none have described what behaviour change techniques were used to change physicians’ imaging behaviour. This limits our understanding of whether any of those interventions were designed to target the known barriers to changing physician ordering practices that were identified in our review. However, it is possible to re-analyse intervention descriptions according to a behaviour change framework to deduce what BCTs were reported as being included in the interventions [19]. This information can provide valuable guidance in the design and evaluation of future implementation interventions that aim to reduce LBP imaging rates. This, combined with the recent attention that unnecessary LBP imaging has received [13], provides a good rationale to conduct a new review that includes more recent studies and to better describe and analyse the interventions of included studies to ascertain what BCTs have been used and in what setting.

Therefore, we aim to produce the first comprehensive review of BCTs used to change physician behaviour to reduce unnecessary LBP imaging and assess whether they are appropriate to target the known barriers identified in our previous qualitative review. While our review will not assess the effectiveness of the interventions, our findings can be used to inform how effectiveness can be evaluated in future systematic reviews.

### Research questions

1. What BCTs (listed in the BCTv1 taxonomy) have been used in interventions designed to change physician behaviour to align with evidence-based guidelines to reduce unnecessary imaging for LBP in both primary care and emergency department settings?
2. Do the identified interventions target known barriers to change?

## Methods

We designed and report the review protocol based on the PRISMA statement [20]. The protocol was registered with PROSPERO (CRD42017072518). Since registering our protocol on PROSPERO, to improve specificity, we decided to only include studies that specifically targeted imaging for LBP rather than studies that targeted improvement of any LBP guideline-based recommendation.

### Data sources and inclusion criteria

Our research librarian (MS) developed a search strategy using a broad range of terms from a previous search strategy [8] related to back pain, diagnostic imaging and relative interventions combined with additional terms for primary and emergency care settings (Additional file 1). Using this strategy, we searched EMBASE, Ovid (Medline), CINAHL and Cochrane CENTRAL from inception to February 1, 2021. We hand-searched references from six relevant systematic reviews [7–10, 21, 22], conducted forward and backward citation tracking of all included studies in our review, and emailed several content experts in the field of imaging and LBP to identify any additional studies our electronic and hand searches may have missed.

Studies were eligible if they were published in English and met the inclusion criteria detailed in Table 1. We excluded studies that targeted patients or the public directly (e.g., mass media campaigns) and interventions that were designed to improve adherence to other aspects of LBP guidelines without targeting imaging.

**Table 1:** Inclusion criteria

Category	Description
**Type of study**	**Include:** • Randomised or cluster randomised trials • Non-randomised controlled trials • Interrupted time-series design • Controlled before-after studies • Uncontrolled before-after studies • Protocols or papers describing interventions **Exclude:** • Non-peer reviewed, unpublished studies
**Types of participants**	**Include:** • Studies that aim to change the behaviour of GPs or emergency department physicians who treat patients with LBP **Exclude:** • Studies aiming to change the behaviour of other health care providers (physiotherapists, chiropractors) using LBP guidelines • Studies aiming to change the behaviour of GPs or ED physicians treating other patient populations (e.g., arthritis, fibromyalgia, generic chronic pain, neck pain, thoracic spinal pain)
**Types of interventions**	**Include:** • Implementation interventions designed to reduce unnecessary LBP imaging. **Exclude:** N/A
**Types of outcome measures**	We did not include inclusion/exclusion criteria based on outcomes

*N/A* not applicable

### Study selection

All titles identified by the search were combined in Endnote (https://endnote.com) and duplicates removed. Two reviewers (DT, RL) screened the initial set of titles and abstracts, excluding irrelevant studies. Two reviewers (AH, AP, HR) then screened the full texts for eligibility. In cases of disagreement, a third reviewer (HE, JP or AP) was consulted.

### Data extraction

Two reviewers (AH and HR, JT or RL) independently extracted study characteristics including year, country, design, setting, target provider and intervention information using an adapted version of the Template for Intervention Description and Replication (TIDieR) checklist [23]. One reviewer (RL) assessed and extracted data on the use of a theoretical rationale for designing/developing the intervention using the Painter criteria [24], which distinguishes the use of theory into the following categories: (i) informed by theory, (ii) applied theory, (iii) testing theory or (iv) building or creating theory.

### Data synthesis and analysis

To achieve objective 1 (to describe what BCTs have been used in interventions designed to change physician’s imaging behaviour) the content of the intervention and control groups was extracted from the descriptions provided in the source paper or from referenced protocols or intervention development papers where appropriate. We coded published intervention descriptions according to the BCT Taxonomy v1 developed by Michie et al. [25]. This comprehensive taxonomy was based on a synthesis of behaviour change literature and includes 93 individual BCTs. Where studies targeted multiple behaviours (beyond reducing LBP imaging), we used the following assumptions to guide whether the study was included in the qualitative BCT intervention synthesis:

- Studies were included if they targeted multiple behaviours and explicitly stated which strategies were used for imaging (explicitly defined) OR they targeted multiple behaviours and included one or more strategies for all target behaviours (implied).
- Studies were excluded if they targeted multiple behaviours and did not specifically state which strategies were used for imaging.

A coding manual was created based on the BCT taxonomy v1 [25]. The BCT coding framework was piloted by all coders experienced with the BCT Taxonomy (CA, JT, AP, AH) on two studies. During this process, clarifications and examples added to the coding manual by the coders to reflect any assumptions made. Using this first version of the coding manual, interventions were coded independently by two coders. Coding was compared across coders for discrepancies and was resolved by consensus and/or discussed with experts on the team (JM, HE and AMP). There were several instances in which discrepancies were discussed with the experts on the team and further rules were added to the codebook for consistency and clarity. These instances largely related to situations in which there was a lack of explicit information to allow for coding a BCT without making assumptions. The team agreed to apply a conservative coding approach in most instances and added exception rules where applicable (see Additional file 2). Using the final version of the codebook, BCT use across studies, along with their associated taxonomy hierarchies, were tabulated by setting (primary care or emergency department).

Following this, to achieve objective 2 (to assess whether the identified interventions target known barriers to change), we used the Theory and Techniques Tool (available at: https://theoryandtechniquetool.humanbehaviourchange.org/tool) to map BCTs to their corresponding mechanisms of action in the theoretical domains framework (TDF) [26, 27]. The Theory and Techniques Tool maps 74 BCTs to 26 mechanisms of action (including the 14 TDF domains) and depicts the strength of association (link) with colour, with stronger links represented in green, and weaker links represented in orange. The strength of a link was determined by combining data from a literature synthesis [26] and an expert consensus study [27]. Where BCTs mapped to more than one TDF domain, we only recorded the domain where there was strong evidence of a link between the BCT and that domain. In cases where there was no strong association between a BCT and any of the TDF domains, we recorded a domain with a weak association to the BCT. Finally, using this information we identified which intervention addressed the three TDF domains that were implicated in our 2019 review of physician reported barriers to implement LBP guidelines [18].

## Results

**Fig. 1:**
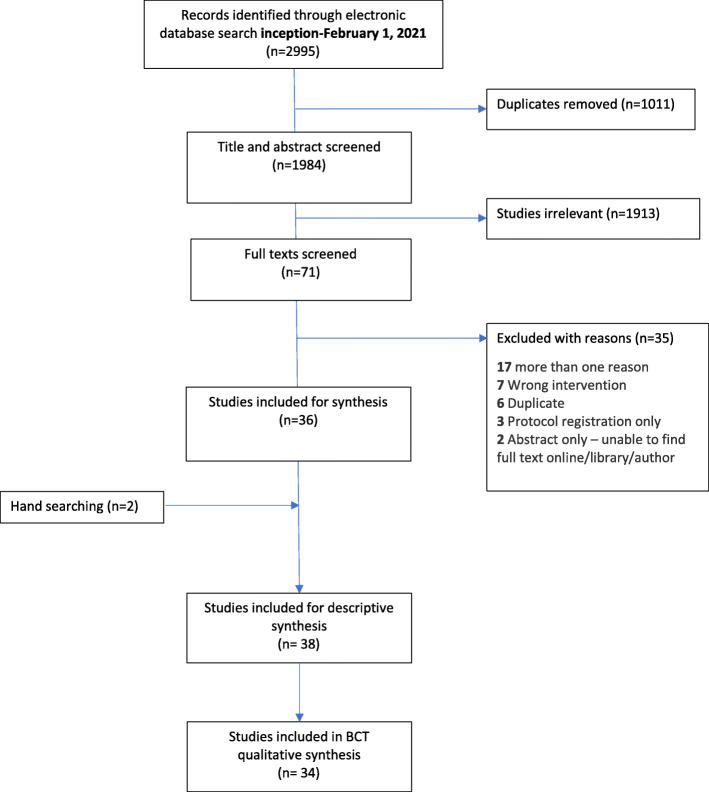
Flow of studies through review using the PRISMA flow diagram

The electronic search (inception to January 2021) identified a total of 2995 citations, 1984 citations after duplicates were removed. From the unique citations, we screened 71 full texts of which 36 were eligible. Hand-searching of five relevant systematic reviews and citation tracking of included studies identified a further 2 studies. Thus, we identified a total of 38 studies that targeted physician behaviour to reduce unnecessary LBP imaging [28–65]. A description of the study identification and selection process is outlined in the PRISMA flow diagram (Fig. 1).

### Description of studies

Nineteen (50%) of the studies were conducted in the last 5 years (between 2015-present) in both primary care (82%) [30–38, 40–42, 44–48, 50, 52–60, 62–64] and emergency settings (18%) [28, 29, 39, 43, 49, 51, 61, 65] in the US (*n* = 17) [28, 30, 33, 35, 38, 39, 41–43, 46, 47, 54, 56, 59, 62, 64], the UK (*n* = 8) [31, 32, 40, 45, 50, 53, 55, 61], Australia (*n* = 6) [7, 44, 48, 49, 52, 58], Canada (*n* = 3) [29, 34, 51], the Netherlands (*n* = 2) [60, 63], Finland (*n* = 1) [57] and Germany (*n* = 1) [65]. Study designs included RCTs/Cluster RCTs (*n* = 12) [31–33, 36, 45, 48, 53, 55, 56, 63–65], Step-wedged RCTs (*n* = 3) [43, 47, 49], interrupted time series (ITS) (*n* = 8) [28, 34, 38, 40–42, 50, 52], controlled before-after studies (CBA) (*n* = 2) [35, 60], uncontrolled before and after studies (UBA) (*n* = 10) [29, 30, 37, 39, 46, 51, 58, 59, 61, 62] and three non-experimental studies (describing either the development (*n* = 2) [44, 57] or post-implementation (*n* = 1) [54] of an eligible intervention). Four of the 38 studies met the Painter criteria for use of theory, with three studies classified as being informed by theor y[44, 48, 49] and one study classified as testing theory [36]. All studies are described in Table 2.

**Table 2:** Description of included studies

Study (year) Country	Target behaviour	Design (control group)	Description of intervention: components, provider and dose	Intervention design: rationale, behavioural theory (Painter criteria*)
**Studies targeting LBP imaging behaviour only/studies targeting multiple behaviours where BCTs that target LBP imaging can be isolated**				
**Target provider (setting): General practitioners (community setting)**				
Fine 2017 [34] Canada	x-ray, CT or MRI	ITS No intervention	**Components:** a) Policy change (once) **Provider:** a) Government	***Rationale:*** Yes. Government withdrew funding for uncomplicated LBP imaging ***Painter****:* No theory
Kullgren 2018 [47] US	x-ray, CT or MRI	Stepped wedge cluster RCT No intervention	**Components:** a) GP commitment (performed once) b) Reminders (post-it notes (provided for every LBP patient) and emails (sent weekly)) c) Patient resources (handout) (provided for every LBP patient) **Provider:** a) Study team b) Medical assistants working in the primary care clinics c) Study team	***Rationale:*** Yes. Intervention ‘pre-commitment’ strategy drawn from behavioural economics ***Painter:*** No theory
Fenton 2016 [33] US	x-ray, CT or MRI	RCT Guidelines only	**Components:** a) Communication training (10 min, provided once) **Provider:** a) Study team	***Rationale:*** Yes. Patient requests may be one factor driving overuse of diagnostic tests and thus, patient-centred communication may address their concerns and reduce imaging requests. ***Painter:*** No theory
French 2013 [36] Australia	x-rays	Cluster RCT Guideline only	**Components:** a) Educational workshops (3 h, provided twice) b) DVD of workshop content (given once) **Provider:** a + b) Study team	***Rationale:*** Yes. They use the TDF and a mapping tool to identity BCTs. Previous interview study identifying barriers to behaviour has been published. ***Painter:*** Yes. testing theory where the theoretical framework was specified (TDF) and more than half of the theoretical constructs applied to the intervention were measured and tested (in their 51-item self-developed questionnaire)
Lin 2016 [48] Australia	x-ray, MRI, CT	ITS	**Components:** a) Educational workshops (3 h, provided twice) b) Audit and feedback (provided once) c) Clinical tools (LBP decision making tool and Start Back screening tool) (provided once) **Provider:** a) Study team b) Study team c) Study team	***Rationale:*** Yes. Previous study of theirs and literature advocating a tailored and theoretically informed approach. ***Painter:*** Yes. Informed by theory.
Winkens 1995 [63] NL	x-ray	Cluster RCT No intervention	**Components:** a) Audit and feedback (provided five times) **Provider:** a) Internal medicine specialist	***Rationale:*** Yes. Previously conducted internal surveys suggested a reduction of testing based on routinely provided feedback but a causal relationship has not been found/studied in a trial context. ***Painter:*** No theory
Jackson 2005 [42] US	x-rays	ITS No intervention	**Components:** a) Educational materials (national guidelines) **Provider:** a) Government	**Rationale:** No ***Painter:*** No theory
Graves 2018 [38] US	x-ray, CT or MRI	ITS No intervention	**Components:** a) Policy change **Provider:** a) Government	***Rationale:*** Yes, government introduced a utilisation review policy ***Painter****:* No theory
Hollingworth 2002 [40] UK	LBP radiology	ITS No intervention	**Components:** a) Educational materials (guidelines) **Provider:** a) Government	***Rationale:*** No ***Painter****:* No theory
Ip 2014 [41] US	Lumbar spine MRI	ITS No intervention	**Components:** a) Decision support (provided for every lumbar MRI request) b) Audit and feedback (provided quarterly) c) Soft stop (peer to peer consultation) (provided every time CDS recommendation to not image was ignored) **Provider:** a) Computerised physician order entry b) Study team c) Radiology dept	***Rationale:*** No ***Painter****:* No theory
Matowe 2002 [50] UK	x-ray	ITS No intervention	**Components:** a) Education materials (guidelines) (provided once) **Provider:** a) Study team	***Rationale:*** Yes, they state that passive dissemination of guidelines has been effective in reducing referrals from primary care and that it would be highly cost-effective (with references). ***Painter****:* No theory
Robling 2002a [55] UK	MRI	Cluster RCT (two sequential trials) Guideline only	**RCT 1** **Components:** a) Change to ordering method (provided throughout study duration) **Provider:** a) Radiology department	***Rationale***: Previous literature on referral method; they state that a multifaceted approach to education may be the most effective ***Painter****:* No theory
Robling 2002b [55] UK	MRI	Cluster RCT (two sequential trials) Guideline only	**RCT 2** **Components:** a) Educational materials (guidelines) (provided once) plus either b) Further education (practice-based seminar) (provided once) or c) Audit and feedback (provided once) or d) Both b and c or e) Nothing (apart from the guidelines) **Provider:** a) Study team b) Academic GP and researcher; c) Not specified d) N/A e) N/A	***Rationale***: Previous literature on referral method; they state that a multifaceted approach to education may be the most effective ***Painter****:* No theory
Eccles 2001 [32] UK	x-ray	Cluster RCT Guideline Only	**Components:** a) Educational materials (guidelines) (provided once) plus either b) Additional education (educational messages) (provided twice) or c) Audit and feedback (provided for every lumbar x-ray) **Provider:** a) GPs and Radiologists; b) Radiology dept c) Study research team	***Rationale***: Yes—previous evidence, saying that’ ‘Oxman and colleagues have reviewed the effectiveness of interventions. Specific prompts at the time of consultation are powerful strategy and have been shown to alter GPs’ behaviour. ***Painter****:* No theory
Kerry 2000 [45] UK	x-ray	Cluster RCT No intervention	**Components:** Educational materials (guidelines) (provided twice) + audit and feedback (provided once) **Provider:** a) Study team b) Study team	***Rationale***: No ***Painter****:* No theory
Oakeshott 1994 [53] UK	x-ray	Cluster RCT No intervention	**Components:** Educational materials (posted guidelines) **Provider:** Study team **Dose:** Provided once	***Rationale***: No ***Painter****:* No theory
Morgan 2019 [52] Australia	X-ray, CT	ITS No intervention	**Components:** Audit and feedback (given once) + ongoing access to a prescription pad and online decision support tool **Provider:** a) Study team b) NPS MedicineWise c) Study team and the George Institute	***Rationale***: Yes, previous evidence and literature ***Painter****:* No theory
Zafar 2019 [64] US	MRI	Cluster RCT Historic control	**Components:** a) Audit and feedback (provided every 4-6 months) or b) Real-time decision support (provided for every lumbar MRI) or c) Both a and b **Provider:** a) Unclear b) Computerised physician order entry system c) As above	***Rationale***: Yes, previous evidence and literature ***Painter****:* No theory
Jenkins 2018 [44] Australia	None	Development of intervention, not looking at imaging outcomes	**Components:** a) Educational workshop (provided once) b) Educational materials provided once) c) Decision support (provided for every LBP patient) d) Patient education materials (provided for every LBP patient without indication for imaging) **Provider:** a) Study team b) Study team c) Study team (used by GP) d) Study team (given to patients by GP)	***Rationale***: Yes, previous evidence and literature ***Painter****:* Yes, informed by theory
Wang 2020 [62] USA	MRI	UBA No control	**Components:** a) Educational presentations (provided once in person and/or virtual) **Provider:** a) Study team	**Rationale:** No **Painter:** No theory
Fried 2018 [37] USA	MRI	UBA No control	**Components:** a) Included a simple epidemiologic statement in lumbar MRI imaging reports **Provider:** a) Statement developed by study team	**Rationale:** Yes, previous research **Painter:** No theory
Klein 2000 [46] USA	CT, MRI	UBA No control	**Components:** a) Educational materials (1-page summary guideline was developed to preface a 16-page detailed guideline) b) Continuing medical educational presentations (provided once, in-person in a large group session or small group session, or via audiotape) c) Clinical champion **Provider:** a) A multi-disciplinary team of practitioners b) A multi-disciplinary team of practitioners b) Rheumatologist	**Rationale:** No **Painter:** No theory
Powell 2019 [54] USA	CT, MRI	Non-experimental Not applicable	**Components:** a) Decision support—nondenial prior authorisation b) Peer-to-peer consultation **Provider:** a) Computerised prior authorisation system b) Board-certified radiologist	**Rationale:** No **Painter:** No theory
Solberg 2010 [59] USA	MRI	UBA No control	**Components:** a) Decision support (provided for every lumbar MRI request) **Provider:** a) Electronic medical record	**Rationale:** Yes, literature and previous research **Painter:** No theory
Suman 2018 [60] NL	X-ray, CT, MRI	CBA No intervention	**Components:** a) Multi-disciplinary continuing medical education training in an evidence-based guideline for LBP was developed in the Netherlands in 2010 including online and offline supplemental educational materials **Provider:** a) Study team	**Rationale:** Yes, literature and previous research **Painter:** No theory
Chen 2020 [30] USA	X-ray, CT, MRI	UBA No control	**Components:** a) Decision support (provided for every lumbar MRI request) **Provider:** a) Electronic medical record	**Rationale:** Yes, literature and previous research **Painter:** No theory
Simula 2019 [57] FIN	Unknown	Intervention development	**Components:** a) Patient education materials (intended for LBP patients without indication for imaging) **Provider:** a) Study team (to be given to patients by GP)	**Rationale**: Yes, previous evidence and literature **Painter**: Yes, informed by theory
Slater 2014 [58] AUS	Unknown	Prospective cohort Non-experimental	**Components:** a) Clinical education programme (based on national and international clinical practice guidelines) consisting of 5 modules and detailed case studies/patient vignettes **Provider:** a) Study team (interprofessional team)	**Rationale:** No **Painter:** No theory
Freeborn 1997 [35] USA	X-ray, CT, MRI	CBA No intervention	**Components:** a) Education (disseminate clinical practice guidelines) b) Decision support aid (flow diagram) b) Feedback on performance **Provider:** a) Team of specialist colleagues b) Study team c) Study team	**Rationale:** No **Painter:** No theory
Jarvik 2015 [43] US	Spine related relative value units	Stepped-wedge cluster RCT No intervention	**Components:** a) Passive education (provided throughout study duration) **Provider:** a) Study team (through EHR systems)	***Rationale:*** Yes. Previous literature and pilot study ***Painter:*** No theory
**Target provider (setting): ED physicians (hospital setting)**				
Min 2017 [51] Canada	x-ray, CT or MRI	UBA No control	**Components:** a) Decision support (provided for every Lumbar image request) b) Educational materials (provided once) c) Patient materials (provided for every LBP patient not imaged) **Provider:** a) Computerised physician order entry system b) Study team c) Provided by a physician (developed by study team)	***Rationale***: Yes, they cite that previous evidence has shown CDS to be effective in outpatients to modify clinician behaviour, but efficacy in ED is yet to be established ***Painter****:* No theory
Chandra 2017 [29] Canada	x-ray	UBA No control	**Components:** a) Educational seminar (provided once) b) Educational videocast (provided once) c) Educational materials (posters) (provided twice) **Provider:** a), b), c) and d) Study team	***Rationale***: No ***Painter****:* No theory
Tracey 1994 [61] UK	x-ray	UBA No control	**Components:** a) Audit and feedback (provided once) b) Educational materials (guidelines) (provided once) c) Educational seminar (provided once) d) Change to ordering process (provided every time a lumbar image was ordered) **Provider:** a), b) and c) Study team d) Radiology dept	***Rationale***: Yes, to develop and introduce their own more detailed guidelines since compliance is more likely when staff are responsible for their development and introduction ***Painter****:* No theory
Baker 1987 [28] US	x-ray	ITS No intervention	**Components:** a) Change to order form (provided every time a lumbar image was ordered) **Provider:** a) Study team	***Rationale***: No ***Painter****:* No theory
**Studies targeting multiple behaviours**				
**Target provider (setting): General practitioners (community setting)**				
Dey 2004 [31] UK	x-ray	Cluster RCT No intervention	**Components:** a) Educational outreach (provided once) b) Ongoing access to fast track physio and back clinic **Provider:** a) Study team b) Usual clinical teams	***Rationale:*** Yes. Previous Cochrane review suggests that educational outreach may be effective for modifying professional behaviour. Fast track triage and physio was offered to avoid referral to secondary care with out of date views on LBP management. ***Painter:*** No theory (in the discussion, they state that the educational outreach was based on theoretical models and give a reference. The reference refers to the ‘elaboration likelihood model of persuasion’). There is no other mention of theory and no measures of constructs.
Schectman 2003 [56] US	x-ray, CT or MRI	Cluster RCT No intervention	**Components:** a) Education session (90 min, provided once) b) Patient materials (provided for every LBP patient) c) Audit and feedback (provided twice) d) Written reminders (provided twice) **Provider:** a) Recognised clinical leaders at each of the respective institutions b) Developed by physicians, a health services researcher, and an expert in patient education materials c) Study team d) Study team	***Rationale:*** No ***Painter:*** No theory
**Target provider (setting): ED physicians (hospital setting)**				
Haig 2019 [39] US	CT, MRI	UBA No control	**Components:** a) Education (email, provided weekly) b) Questionnaire (ongoing access) c) Order sheet (ongoing access) d) Ongoing access to fast track access to psychiatry and physical therapy **Provider:** a) Principal investigator at site b) ED triage staff c) ED triage staff d) N/A	***Rationale:*** Yes. Previous literature and evidence ***Painter:*** No theory
Burggraf 2019 [65] Germany	x-ray, MRI, CT	Cluster RCT No intervention	**Components:** a) Educational workshop (one day, provided once) b) Ongoing access to online educational materials **Provider:** a) Study team b) Study team	***Rationale:*** Yes. Previous evidence and literature ***Painter:*** No theory
Machado 2018 [49] Australia	x-ray, MRI, CT	Stepped-wedge cluster RCT No intervention (all clusters will ultimately end up with the intervention)	**Components:** a) Education session (40–60 min, provided once with some booster sessions) b) Educational outreach (provided once) b) Audit and feedback (provided monthly) c) Patient resources (provided for every LBP patient) **Provider:** a) Local opinion leaders b) Clinical educators c) Study team d) The Agency for Clinical Innovation	***Rationale:*** Yes. Large deviations in Australian EDs make them ideal to trial a new model of LBP. The ACI model of care was jointly developed by policy makers, clinicians, consumers and researchers and translated high-quality evidence into key practice messages. ***Painter:*** Yes, informed by theory (the KTA framework)

### Description of interventions

Of the 38 studies, we excluded 4 studies [31, 39, 49, 56] from our BCT qualitative synthesis since they targeted multiple aspects of LBP guideline-recommended behaviours and it was not possible to isolate which BCTs were targeting imaging only. The remaining 34 studies either i) solely targeted a reduction in imaging, ii) used the same intervention strategy for all of its target behaviours or iii) specified which BCTs targeted LBP imaging behaviour. We identified 18 BCTs across the 34 studies (see Table 3); the most common techniques were ‘4.1 instruction on how to perform the behaviour’ (*n* = 31 interventions; 26 studies) [29, 32, 33, 35, 36, 40–42, 44–48, 50, 52, 53, 55, 57–65], defined as ‘advise or agree on how to perform the behaviour’, and ‘9.1 credible source’ (*n* = 21 interventions; 18 studies) [29, 30, 32, 35, 36, 40–42, 45, 46, 48, 50, 53, 55, 58, 59, 62, 63], defined as ‘present verbal or visual communication from a credible source in favour of or against the behaviour’, ‘7.1 prompts/cues’ (*n* = 14 interventions; 14 studies) [29, 30, 32, 35, 41, 42, 44, 46, 53, 54, 57, 59, 61, 64] defined as ‘introduce or define environmental or social stimulus with the purpose of prompting or cuing the behaviour’, and ‘2.2 feedback on behaviour’ (*n* = 13 interventions; 12 studies) [30, 32, 33, 35, 41, 45, 48, 52, 55, 61, 63, 64], defined as ‘monitor and provide informative or evaluative feedback on performance of the behaviour’, and ‘12.5 adding objects to the environment’ (*n* = 11 interventions, 11 studies) [30, 35, 36, 44, 47, 48, 51, 52, 54, 57, 59] defined as ‘adding objects to the environment in order to facilitate the behaviour’. In these studies, 4.1 commonly referred to education based around a guideline or the provision of a guideline either delivered via post or in person or virtually via webinar, 9.1 commonly referred to the guideline being produced by a reputable/credible institution (not a society) or the guideline was verbally endorsed via video or in person by a reputable peer, 7.1 commonly referred to an automated decision support tool (implemented within a computer pop-up window) or as a hard copy summary of the guidelines/decision aid on when to image (implemented as a poster, laminated document or education booklet), 2.2 commonly referred to the use of audit and feedback in which a feedback report was delivered via email or post to physicians providing them with data on their imaging use and 12.5 commonly referred to decision support tools or education booklets that could be given to patients to help them understand their diagnosis and why imaging was not necessary. The highest number of BCTs included in a single study was 8 [36], while 5 interventions (4 studies) [34, 38, 55, 60] included only a single BCT.

**Table 3:** BCTs identified within study intervention descriptions

	Goals & planning		Feedback and monitoring			Shaping knowledge	Natural consequences	Comparison of behaviour		Association	Repetition / substitution	Comparison of outcomes	Antecedents				Totals
	1.1. Goal setting (behaviour)	1.9. Commitment	2.2. Feedback on behaviour	2.3. Self-monitoring of own behaviour	2.7. Feedback on outcome(s) of behaviour	4.1. Instruction on how to perform the behaviour	5.1. Information about health consequences	6.1. Demonstration of the behaviour	6.2. Social comparison	7.1. Prompts/cues	8.1. Behavioural practice/rehearsal	9.1. Credible source	12.1. Restructuring the physical environment	12.2. Restructuring the social environment	12.5. Adding objects to the environment	13.2. Framing / reframing	
**Primary Care**																	
**Eccles 2001 Group 1** [32]			x			x			x			x					**4**
**Eccles 2001** [32] **Group 2**						x				x		x					**3**
**Eccles 2001** [32] **Group 3**			x			x			x	x		x					**5**
**Fenton 2016** [33]			x			x		x			x						**4**
**Fine 2017** [34]													x				**1**
**French 2013** [36]				x		x	x	x			x	x			x	x	**8**
**Graves 2018** [38]													x				**1**
**Hollingworth 2002** [40]						x						x					**2**
**Ip 2014** [41]			x			x			x	x		x	x	x			**7**
**Jackson 2005** [42]						x						x					**2**
**Jenkins 2018** [44]						x		x		x					x		**4**
**Kerry 2000** [45]			x			x	x					x					**4**
**Kulgreen 2018** [47]	x	x				x				x					x		**5**
**Lin 2016** [48]			x			x	x	x			x	x			x		**7**
**Matowe 2002** [50]						x						x					**2**
**Morgan 2019** [52]			x			x			x						x		**4**
**Oakeshott 1994** [53]						x				x		x					**3**
**Robling 2002a** [55]													x				**1**
**Robling 2002b** [55] **Group 1**						x						x					**2**
**Robling 2002b** [55] **Group 2**			x			x			x								**3**
**Robling 2002b** [55] **Group 3**			x			x			x			x					**4**
**Robling 2002b** [55] **Group 4**						x											**1**
**Winkens 1995** [63]			x			x						x					**3**
**Zafar 2019** [64]			x			x			x	x							**4**
**Wang 2020** [62]						x		x				x					**3**
**Fried 2018** [37]					X		X										**2**
**Klein 2000** [46]						x						x					**3**
**Powell 2019** [54]										x				x	x		**3**
**Solberg 2010** [59]						x				x		x			x		**4**
**Suman 2018** [60]						X											**1**
**Chen 2020** [30]										x		x			x		**3**
**Simula 2019** [57]						x		x		x					x		**4**
**Slater 2014** [58]						x		x			x	x					**4**
**Freeborn 1997** [35]			x			x			x	x		x			x		**6**
**Emergency Department**																	
**Baker 1987** [28]													x	x			**2**
**Chandra 2017** [29]						x				x		x					**3**
**Min 2017** [51]													x		x		**2**
**Tracey 1994** [61]			x			x	x			x			x				**5**
**Jarvik 2015** [43]					x		x										**2**
**Burggraf 2019** [65]						x		x			x						**3**
**Totals**	**1**	**1**	**13**	**1**	**2**	**31**	**6**	**8**	**8**	**14**	**5**	**21**	**7**	**3**	**11**	**1**	**133**

Greater than 50% of studies used a BCT that targeted one of the following TDF domains Knowledge (*n* = 34 interventions; 27 studies) [29, 32, 33, 35–37, 40–48, 50, 52, 53, 55, 57–65], Skills (*n* = 32 interventions; 27 studies) [29, 31–33, 35, 36, 40–42, 44–48, 50, 52, 53, 55, 57–65] and Beliefs about capabilities (*n* = 32; 27 studies) [29, 31–33, 35, 36, 40–42, 44–48, 50, 52, 53, 55, 57–65]. Following these, the domains of environmental context and resources (*n* = 22 interventions; 21 studies) [28–30, 32, 34–36, 38, 41, 44, 47, 48, 51–55, 57, 59, 61, 64], social/professional role/identity (*n* = 22; 18 studies) [29, 30, 32, 35, 36, 40–42, 45, 46, 48, 50, 53, 55, 58, 59, 62, 63], reinforcement (*n* = 16; 13 studies) [29, 32, 33, 41, 45, 47, 48, 52, 53, 55, 61, 63, 64], memory (*n* = 14; 13 studies) [29, 30, 32, 35, 41, 44, 47, 53, 54, 57, 59, 61, 64], social influence (*n* = 10; 8 studies) [28, 32, 35, 41, 52, 54, 55, 64] were often targeted. The only domain that was not targeted by interventions in the included studies was optimism. Table 4 provides a detailed mapping of interventions BCTs to TDF domains.

**Table 4:** TDF domains targeted by each study through the use of BCTs

	Knowledge	Skills	Social/ professional role / identity	Beliefs about capabilities	Optimism	Beliefs about consequences	Reinforce-ment	Intentions	Goals	Memory, Attention and Decision Processes	Environmental context and resources	Social influences	Emotion	Behavioural Regulation
**Primary Care**														
**Eccles 2001** [32] **Group 1**	x	x	x	x			x					x		
**Eccles 2001** [32] **Group 2**	x	x	x	x			x			x	x			
**Eccles 2001** [32] **Group 3**	x	x	x	x			x			x	x	x		
**Eccles 2001** [32] **Group 4**	x	x	x	x										
**Fenton 2016** [33]	x	x		x			x							
**Fine 2017** [34]											x			
**French 2013** [36]	x	x	x	x		x		x			x		x	x
**Graves 2018** [38]											x			
**Hollingworth 2002** [40]	x	x	x	x										
**Ip 2014** [41]	x	x	x	x			x			x	x	x		
**Jackson 2005** [42]	x	x	x	x										
**Jenkins 2018** [44]	x	x		x						x	x			
**Kerry 2000** [45]	x	x	x	x		x	x	x						
**Kulgreen 2018** [47]	x	x		x			x	x	x	x	x			x
**Lin 2016** [48]	x	x	x	x			x				x			
**Matowe 2002** [50]	x	x	x	x										
**Morgan 2019** [52]	x	x		x			x				x	x		
**Oakeshott 1994** [53]	x	x	x	x			x			x	x			
**Robling 2002a** [55]											x			
**Robling 2002b** [55] **Group 1**	X	x	x	x										
**Robling 2002b** [55] **Group 2**	X	x		x			x					x		
**Robling 2002b** [55] **Group 3**	X	x	x	x			x					x		
**Robling 2002b** [55] **Group 4**	X	x		x										
**Winkens 1995** [63]	X	x	x	x			x							
**Zafar 2019** [64]	X	x		x			x			x	x	x		
**Wang 2020** [62]	X	x	x	x										
**Fried 2018** [37]	X					x		x						
**Klein 2000** [46]	X	x	x	x										
**Powell 2019** [54]										x	x	x		
**Solberg 2010** [59]	X	x	x	x						x	x			
**Suman 2018** [60]	X	x		x										
**Chen 2020** [30]			x							x	x			
**Simula 2019** [57]	x	x		x						x	x			
**Slater 2014** [58]	X	x	x	x										
**Freeborn 1997** [35]	X	x	x	x						x	x	x		
**Jarvik 2015** [43]	x					x		x						
**Emergency Dept.**														
**Baker 1987** [28]											x	x		
**Chandra 2017** [29]	x	x	x	x			x			x	x			
**Min 2017** [51]											x			
**Tracey 1994** [61]	x	x		x		x	x	x		x	x			
**Burggraf 2019** [65]	x	x		x										
**Totals**	34	32	22	32	0	5	16	6	1	14	22	10	1	2

### Description of how studies targeted the TDF domains through the use of BCTs (see Additional file 3)

Many of the identified BCTs target more than one TDF domain according to Michie et al.’s guide (the Theory and Techniques Tool, https://theoryandtechniquetool.humanbehaviourchange.org/tool, [26, 27]). For example, 4.1 instruction on how to perform the behaviour maps to three TDF domains (knowledge, skills and beliefs about capabilities) meaning that using the BCT of 4.1 may help to overcome barriers at one or all three of these domains. Thus, where applicable, according to the Theory and Techniques tool, TDF domains that were frequently targeted together have been described together below. As stated in the methods, for this section of the synthesis, we have only reported a link between a BCT and mechanism of action where it has strong evidence. In cases where there were no links with strong evidence, we have reported that and cited the links with weak evidence (see the Theory and Techniques tool).

#### Knowledge, skills and beliefs about capabilities

Of the studies targeting the domains of knowledge, skills and beliefs about capabilities, 31 interventions (26 studies) used the BCT 4.1 ‘instruction on how to perform the behaviour’ by providing education on imaging guidelines either via active (face-to-face education sessions or webinars) or passive dissemination (email or postal delivery of the guideline). Nine of these interventions (6 studies) also provided education on topics related to imaging such as indicators for imaging, consequences of inappropriate imaging (BCT 5.1 ‘information about health consequences’) and alternatives to imaging, while 3 interventions (3 studies) provided education that was not specific to a guideline (such as communication skills training). Five studies explicitly used the BCT 8.1 ‘behavioural practice/rehearsal’ through interactive practice (*n* = 2), role play (*n* = 2), and skill rehearsal (*n* = 1). Six studies used additional strategies to specifically target beliefs about capabilities which included the use of patient scenarios (*n* = 2) (BCT 8.1) and demonstration of desired behaviours (BCT 6.1 ‘demonstration of the behaviour’) including how to use new clinical tools (such as decision support tools) in a consultation or how to prescribe an activity programme instead of ordering an image (*n* = 4).

#### Social/professional role and identity

There is weak evidence that 21 interventions targeted this domain (18 studies) with the BCT 9.1 ‘credible source’. The majority implemented this BCT by using guidelines produced by a recognised body/credible source (*n* = 17 interventions, 14 studies). The remaining studies implemented this BCT through accreditation of their training with a professional body (*n* = 1) or by using experts to deliver education/training (*n* = 4 interventions, 4 studies).

#### Beliefs about consequences; intentions

Five studies targeted the domains beliefs about consequences and intentions through the BCT 5.1 ‘information about health consequences’ by focusing on the negative consequences of imaging (*n* = 3) and providing education on radiation exposure (*n* = 2). One additional study targeted ‘intentions’, along with the domain of goals, through goal setting (BCT 1.1 ‘goal setting (behaviour)’).

#### Reinforcement

There is weak evidence that 17 interventions (14 studies) targeted this domain. Eleven of these interventions (10 studies) used feedback from an audit or assessment (BCT 2.2 ‘feedback on behaviour’) and 3 studies combined feedback with use of reminders (BCT 7.1 ‘prompts/cues’). 6 interventions (5 studies) used reminders only and one of these combined reminders with pre-commitment (BCT 7.1).

#### Environmental context and resources; memory, attention and decision processes

Seventeen interventions (16 studies) targeted environmental context and resources through either a single or multiple BCTs. 13 interventions (12 studies) used the BCT 7.1 ‘prompts/cues’ through decision tools (*n* = 6 interventions/studies) and reminders (*n* = 7 interventions, 6 studies). 5 interventions (5 studies) used the BCT 12.5 ‘adding objects to the environment’ with patient materials, 1 intervention (1 study) used the BCT 12.1 ‘restructuring the physical environment’ through restricted reimbursement, and 5 interventions (5 studies) used the BCT 12.2 ‘restructuring the social environment’ by implementing soft, medium or hard stops to the image ordering process. A soft stop included changes to the ordering process to require additional details, a medium stop included a peer to peer conversation as part of the ordering process, and a hard stop included either sign off from a consultant to order an image or an inability to order an image without the presence of red flags. The studies described above that used reminders and/or decision tools (BCT 7.1 ‘prompts/cues’) will have also targeted the domain of memory, attention and decision processes (*n* = 9 interventions, 8 studies).

#### Social influences

Eight interventions (6 studies) targeted social influences though the BCT 6.2 ‘social comparison’, namely through peer comparison, feeding back information on how an individual’s or practices’ behaviour compared to those of their peers or a specialist in the field (*n* = 7 interventions, 5 studies).

#### Behavioural regulation and emotion

One study targeted these domains with the BCT 2.3 ‘self-monitoring of own behaviour’. There is weak evidence that one study targeted the domain Behavioural regulation with the BCT 1.1 ‘goal setting (behaviour)’. There is weak evidence that one study targeted the domain Emotion with the BCT 13.2 ‘framing/reframing’.

## Discussion

### Summary

Searching up to February 1, 2021, we found 38 studies evaluating interventions designed to reduce unnecessary LBP imaging. Unlike previous reviews in this field, this review did not explore intervention effectiveness and instead, focused solely on developing an understanding of these implementation interventions designed to reduce LBP imaging. Thus, this review identifies what BCTs have been used to reduce LBP imaging for the first time and, through mapping these BCTs to corresponding TDF domains, provides unique insight into the interventions’ mechanisms of action.

This review highlights that the majority of studies in this field have used education to target the domains of knowledge, skills and beliefs about capabilities to bring about a change in LBP imaging behaviour. Just over half of the studies used reminders, decision tools or changes to the image ordering process to target the domain environmental context and resources. Lastly, for just over half of the studies, inconclusive evidence from the Theory and Techniques Tool suggests that by using feedback and reminders, studies will have targeted the domain of reinforcement, and through using materials from a credible source, will have targeted the domain of social/professional role and identity.

### Strengths

We utilised a comprehensive search strategy across a range of databases and included hand searching references from relevant articles to identify studies. Our primary analysis was guided by the validated and widely used BCT taxonomy, enabling readers to easily draw comparisons to other studies and build on the current work. Our secondary analysis was informed by the Theory and Techniques Tool, developed by international leaders in the field of behaviour change. We iteratively developed a BCT code book, detailing all coding assumptions and rules, to ensure consistency between coders and to provide transparency to readers. Lastly, we used at least two reviewers to code BCTs and TDF domains, utilising a third reviewer where needed to resolve any ambiguity or discrepancies.

### Limitations

While the search strategy we used was comprehensive and we did identify additional studies from hand-searching the references of other relevant systematic reviews, it is still possible that some studies were missed. Further, our conservative coding may have resulted in missing BCTs that more liberal coders may have included; better reporting of study interventions would have allowed us to more accurately capture all BCTs.

### Implications for research

This review highlighted that nearly all study interventions were poorly designed, failing to use theory to inform intervention development and preventing assessments of fidelity and process outcomes. Despite international recognition that behaviour change interventions should be underpinned by theory, only 6 (21%) of our included studies could be classified as having utilised some element of theory to inform their intervention design. This finding is in line with a large systematic review that explored the use of theory in the design of guideline dissemination and implementation strategies [66]. Davies et al. found that only 22.5% of studies (*n* = 235) utilised theory in some way and noted there was inadequate justification for intervention choice [66]. A lack of theory in behaviour change intervention design has been reported in other fields, for example, a systematic review of interventions designed to improve self-management of LBP and arthritis found that only 12% of studies utilised theory [67]. These findings suggest that BCTs are frequently selected based on availability/ease of delivery/cost, as opposed to being actively selected to target known barriers to the behaviour. A recent systematic review synthesised 14 qualitative studies with physicians worldwide and found high-level evidence that 3 TDF domains influenced LBP imaging behaviour: (i) Social influence (patient demand), (ii) Beliefs about consequences (lack of ability to explain why an image is not needed) and (iii) Environmental context and resources (lack of time) [18]. However, we found less than a quarter of our included studies targeted Social influence and even fewer targeted Beliefs about consequences. Thus, highlighting the need to use more robust methods, guided by theory, to select BCTs that target LBP image ordering behaviour.

## Conclusions

This is the first systematic review to synthesise interventions designed to reduce physician LBP image ordering behaviour to identify intervention BCTs. We found the most frequently used BCTs were ‘instruction on how to perform the behaviour’ (most commonly through education based on imaging guidelines), ‘feedback on behaviour’ (most commonly through audit and feedback), ‘prompts and cues’ (most commonly through automated decision support pop-ups or hard-copy guideline-based reminders/decision aids on when to image) and ‘credible source’ (guidelines produced from a credible source or endorsed by a reputable peer). Consequently, the most frequently targeted TDF domains were knowledge, skills and beliefs about capabilities. Importantly, this review identified a lack of theory and rationale in the selection of intervention BCTs with the majority of studies failing to include BCTs to target known physician-reported barriers. Future work should utilise theory to develop interventions specifically targeting known barriers to reducing LBP image ordering.

## Supplementary Information

**Additional file 1.**

**Additional file 2.**

**Additional file 3.**

## Data Availability

Data sharing is not applicable to this article as no datasets were generated or analysed during the current study.

## Abbreviations

BCT: Behaviour change techniques
ITS: Interrupted time series
LBP: Low back pain
PRISMA: Preferred Reporting Items for Systematic Reviews and Meta-Analyses
PROSPERO: International Prospective Register of Systematic Reviews
RCT: Randomised controlled trial
TDF: Theoretical domains framework
UBA: Uncontrolled before and after studies
