# Supplementary material for: What behaviour change techniques have been used to improve adherence to evidence-based low back pain imaging?
Source: Implement Sci. 2021 Jul 2;16:68. doi: 10.1186/s13012-021-01136-w (PMC8254222; doi:10.1186/s13012-021-01136-w)
Supplement: Supplementary file 1 — Additional file 1. [file 13012_2021_1136_MOESM1_ESM.doc]

**Database: Ovid MEDLINE(R) and Epub Ahead of Print, In-Process & Other Non-Indexed Citations and Daily <1946 to February 01, 2021>**

Search Strategy:

--------------------------------------------------------------------------------

1 Primary Health Care/ (79802)

2 physicians, primary care/ (3640)

3 primary healthcare.ti,ab. (6376)

4 (primary adj2 care).ti,ab. (140653)

5 community medicine/ (2036)

6 community health centers/ (7196)

7 exp Community Health Services/ (309005)

8 community.ti,ab. (508891)

9 Ambulatory Care/ (43876)

10 (ambulatory adj (care or setting?)).ti,ab. (11779)

11 physicians' offices/ (1753)

12 clinic?.ti,ab. (363017)

13 physicians, family/ (16485)

14 (family adj (medicine or practice or practitioner? or physician? or doctor?)).ti,ab. (26778)

15 general practice/ (13680)

16 general practitioners/ (8227)

17 (general adj (practice or practioner?)).ti,ab. (36124)

18 (gp or gps).ti,ab. (60600)

19 exp Emergency Service, Hospital/ (82075)

20 Emergency Medicine/ (13828)

21 (emergency adj2 (department? or unit? or room? or physician?)).ti,ab. (126420)

22 (trauma adj2 (centre? or center? or department? or unit?)).ti,ab. (19416)

23 (triage adj2 (centre? or center? or department? or unit?)).ti,ab. (679)

24 ("accident and emergency" or "accident & emergency").ti,ab. (4735)

25 or/1-24 (1492854)

26 Back Pain/ (17819)

27 Low Back Pain/ (22516)

28 (back or backache or backpain).ti,ab. (207791)

29 dorsalgia.ti,ab. (99)

30 exp Spine/ (147793)

31 Lumbosacral Region/ (12724)

32 exp Spinal Diseases/ (126067)

33 (lumbar or lumbosacral or 'lumbo sacral' or spine or spinal).ti,ab. (411256)

34 or/26-33 (687134)

35 diagnostic imaging/ (41448)

36 dg.fs. (1222630)

37 exp radiography/ (1124971)

38 exp magnetic resonance imaging/ (465120)

39 exp image interpretation, computer-assisted/ (551581)

40 imaging.ti,ab. (844750)

41 radiograph*.ti,ab. (220201)

42 radiolog*.ti,ab. (264423)

43 CT.ti,ab. (355009)

44 'computed tomography'.ti,ab. (257592)

45 ('x ray?' or xray?).ti,ab. (368545)

46 (mri or mris).ti,ab. (254236)

47 or/35-46 (2942749)

48 quality improvement/ (26579)

49 program evaluation/ (64224)

50 (program? or programme?).ti,ab. (867880)

51 exp education, continuing/ (61531)

52 staff development/ (9542)

53 Inservice Training/ (20451)

54 ed.fs. (283786)

55 (educat* or teach* or train or training or instruction* or learn*).ti,ab. (1447450)

56 (lecture* or seminar* or presentation* or tutorial* or workshop* or 'work shop' or 'work shops' or webinar*).ti,ab. (477840)

57 academic detailing.ti,ab. (555)

58 patient education as topic/ (86290)

59 exp consumer health information/ (10152)

60 patient acceptance of healthcare/ (48830)

61 'patient education'.ti,ab. (18241)

62 'consumer health'.ti,ab. (1585)

63 exp teaching materials/ (119935)

64 pamphlets/ (3940)

65 (leaflet? or booklet? or poster? or pamphlet?).ti,ab. (38624)

66 information dissemination/ (17406)

67 translational medical research/ (11311)

68 'knowledge translation'.ti,ab. (2829)

69 postal service/ (2287)

70 exp telecommunications/ (97318)

71 exp mass media/ (46017)

72 ((written or printed or oral) adj information).ti,ab. (2321)

73 ((written or printed or oral) adj communication?).ti,ab. (1108)

74 ((media or communication?) adj campaign?).ti,ab. (2608)

75 choosing wisely.ti,ab. (846)

76 marketing.ti,ab. (26782)

77 practice guidelines as topic/ (121897)

78 guideline adherence/ (32974)

79 (guideline? adj2 (adher* or comply* or complies or compliance or disseminat* or distribut*)).ti,ab. (6954)

80 (protocol? adj2 (adher* or comply* or complies or compliance or disseminat* or distribut*)).ti,ab. (2732)

81 (policy adj2 (change? or changing or modifi* or revise or revised or revision? or update?)).ti,ab. (8593)

82 decision support techniques/ (20863)

83 decision support systems, clinical/ (8277)

84 reminder systems/ (3555)

85 'decision support'.ti,ab. (15470)

86 reminder?.ti,ab. (13361)

87 prompt?.ti,ab. (73405)

88 alert?.ti,ab. (31326)

89 algorithm?.ti,ab. (265838)

90 exp clinical audit/ (22465)

91 (audit* adj2 (clinical or medical or record? or chart?)).ti,ab. (6591)

92 chart review*.ti,ab. (44127)

93 feedback/ (30121)

94 feedback.ti,ab. (143308)

95 benchmarking/ (13818)

96 Attitude of Health Personnel/ (124150)

97 exp Health Knowledge, Attitudes, Practice/ (114921)

98 (attitude? adj2 (physician? or resident? or clinician? or provider?)).ti,ab. (5889)

99 (belief? adj2 (physician? or resident? or clinician? or provider?)).ti,ab. (1208)

100 behavio?ral change?.ti,ab. (19813)

101 Practice Patterns, Physicians'/ (61774)

102 Utilization Review/ (8103)

103 utili?ation.ti,ab. (214046)

104 or/48-103 (3805068)

105 exp medical overuse/ (7583)

106 (overuse or 'over use').ti,ab. (11986)

107 (decreas* adj2 (referral* or imaging or radiograph* or radiolog* or CT or 'computed tomography' or 'x ray?' or xray? or mri or mris)).ti,ab. (3302)

108 (reduc* adj2 (referral* or imaging or radiograph* or radiolog* or CT or 'computed tomography' or 'x ray?' or xray? or mri or mris)).ti,ab. (7195)

109 (increas* adj2 (referral* or imaging or radiograph* or radiolog* or CT or 'computed tomography' or 'x ray?' or xray? or mri or mris)).ti,ab. (11825)

110 (unnecessary adj2 (referral* or imaging or radiograph* or radiolog* or CT or 'computed tomography' or 'x ray?' or xray? or mri or mris)).ti,ab. (1309)

111 (appropriate* adj2 (referral* or imaging or radiograph* or radiolog* or CT or 'computed tomography' or 'x ray?' or xray? or mri or mris)).ti,ab. (6389)

112 (inappropriate* adj2 (referral* or imaging or radiograph* or radiolog* or CT or 'computed tomography' or 'x ray?' or xray? or mri or mris)).ti,ab. (566)

113 or/106-112 (41078)

114 exp clinical trial/ (881600)

115 exp clinical trials as topic/ (351833)

116 random*.ti,ab. (1199081)

117 Health Services Research/ (37315)

118 Comparative Study/ (1881482)

119 Controlled Before-After Studies/ (584)

120 Prospective Studies/ (562088)

121 prospective.ti,ab. (599008)

122 (before adj2 after).ti,ab. (286811)

123 (pretest adj posttest).ti,ab. (3593)

124 pre post test*.ti,ab. (1022)

125 pretesting.ti,ab. (1085)

126 repeated measurement?.ti,ab. (9201)

127 repeated measure?.ti,ab. (39034)

128 time series.mp. (34475)

129 (control* adj3 (study or design or trial or matched or group)).mp. (1295753)

130 quasi-experimental.mp. (14529)

131 or/114-130 (4686713)

132 25 and 34 and 47 and (104 or 113) and 131 (734)

**Embase via embase.com**

Search conducted 4 February 2021

| **No.** | **Query** | **Results** |
| --- | --- | --- |
| 1 | 'primary health care'/exp | 176,172 |
| 2 | 'primary healthcare':ab,ti | 7,563 |
| 3 | (primary NEAR/2 care):ab,ti | 186,906 |
| 4 | 'community medicine'/de | 2,800 |
| 5 | 'community care'/de | 60,536 |
| 6 | community:ab,ti | 639,216 |
| 7 | 'ambulatory care'/exp | 51,683 |
| 8 | (ambulatory NEAR/1 (care OR setting$)):ab,ti | 16,787 |
| 9 | clinic$:ab,ti | 587,346 |
| 10 | (family NEAR/1 (medicine OR pratice OR practitioner$ OR physician$ OR doctor$)):ab,ti | 27,970 |
| 11 | 'general practice'/de | 85,856 |
| 12 | 'general practitioner'/de | 102,874 |
| 13 | (general NEAR/1 (practice OR practioner$)):ab,ti | 45,752 |
| 14 | gp:ab,ti OR gps:ab,ti | 86,868 |
| 15 | 'hospital emergency service'/de | 6,075 |
| 16 | 'emergency health service'/de | 103,613 |
| 17 | 'emergency medicine'/de | 41,948 |
| 18 | (emergency NEAR/2 (department$ OR unit$ OR room$ OR physician$)):ab,ti | 195,917 |
| 19 | (trauma NEAR/2 (centre$ OR center$ OR department$ OR unit$)):ab,ti | 25,145 |
| 20 | (triage NEAR/2 (centre$ OR center$ OR department$ OR unit$)):ab,ti | 937 |
| 21 | 'accident and emergency':ab,ti OR 'accident & emergency':ab,ti | 6,006 |
| 22 | #1 OR #2 OR #3 OR #4 OR #5 OR #6 OR #7 OR #8 OR #9 OR #10 OR #11 OR #12 OR #13 OR #14 OR #15 OR #16 OR #17 OR #18 OR #19 OR #20 OR #21 | 1,894,332 |
| 23 | 'backache'/de | 57,830 |
| 24 | 'low back pain'/de | 60,041 |
| 25 | back:ab,ti OR backache:ab,ti OR backpain:ab,ti | 287,368 |
| 26 | dorsalgia:ab,ti | 166 |
| 27 | 'spine'/exp | 206,182 |
| 28 | 'lumbosacral region'/de | 1,358 |
| 29 | 'spine disease'/exp | 259,589 |
| 30 | lumbar:ab,ti OR lumbosacral:ab,ti OR 'lumbo sacral':ab,ti OR spine:ab,ti OR spinal:ab,ti | 547,480 |
| 31 | #23 OR #24 OR #25 OR #26 OR #27 OR #28 OR #29 OR #30 | 991,995 |
| 32 | 'diagnostic imaging'/de | 194,737 |
| 33 | 'radiography'/exp OR radiotherapy:lnk | 1,606,310 |
| 34 | 'nuclear magnetic resonance imaging'/exp | 999,824 |
| 35 | 'computer assisted tomography'/exp | 1,148,057 |
| 36 | imaging:ab,ti | 1,164,443 |
| 37 | radiograph*:ab,ti | 280,968 |
| 38 | radiolog*:ab,ti | 391,917 |
| 39 | ct:ab,ti | 599,517 |
| 40 | 'computed tomography':ab,ti | 321,464 |
| 41 | 'x ray$':ab,ti OR xray$:ab,ti | 423,258 |
| 42 | mri:ab,ti OR mris:ab,ti | 436,792 |
| 43 | #32 OR #33 OR #34 OR #35 OR #36 OR #37 OR #38 OR #39 OR #40 OR #41 OR #42 | 4,161,216 |
| 44 | 'total quality management'/de | 69,390 |
| 45 | 'program evaluation'/de | 15,980 |
| 46 | program$:ab,ti OR programme$:ab,ti | 1,156,921 |
| 47 | 'continuing education'/de | 32,044 |
| 48 | 'in service training'/de | 16,411 |
| 49 | educat*:ab,ti OR teach*:ab,ti OR train:ab,ti OR training:ab,ti OR instruction*:ab,ti OR learn*:ab,ti | 1,919,493 |
| 50 | lecture*:ab,ti OR seminar*:ab,ti OR presentation*:ab,ti OR tutorial*:ab,ti OR workshop*:ab,ti OR 'work shop':ab,ti OR 'work shops':ab,ti OR webinar*:ab,ti | 723,003 |
| 51 | 'academic detailing':ab,ti | 811 |
| 52 | 'patient education'/de | 115,142 |
| 53 | 'consumer health information'/de | 3,966 |
| 54 | 'patient education':ab,ti | 27,909 |
| 55 | 'consumer health':ab,ti | 1,602 |
| 56 | leaflet$:ab,ti OR booklet$:ab,ti OR poster$:ab,ti OR pamphlet$:ab,ti | 66,149 |
| 57 | 'information dissemination'/de | 21,779 |
| 58 | 'knowledge translation':ab,ti | 3,797 |
| 59 | 'mass communication'/exp | 590,457 |
| 60 | ((written OR printed OR oral) NEAR/1 information):ab,ti | 4,159 |
| 61 | ((written OR printed OR oral) NEAR/1 communication$):ab,ti | 1,627 |
| 62 | ((media OR communication$) NEAR/1 campaign$):ab,ti | 3,225 |
| 63 | 'choosing wisely':ab,ti | 1,488 |
| 64 | marketing:ab,ti | 37,467 |
| 65 | 'protocol compliance'/de | 14,966 |
| 66 | (guideline$ NEAR/2 (adher* OR comply* OR complies OR compliance OR disseminat* OR distribut*)):ab,ti | 11,492 |
| 67 | (protocol$ NEAR/2 (adher* OR comply* OR complies OR compliance OR disseminat* OR distribut*)):ab,ti | 4,180 |
| 68 | (policy NEAR/2 (change$ OR changing OR modifi* OR revise OR revised OR revision$ OR update$)):ab,ti | 10,789 |
| 69 | 'decision support system'/exp | 26,916 |
| 70 | 'reminder system'/de | 2,708 |
| 71 | 'decision support':ab,ti | 20,233 |
| 72 | reminder$:ab,ti | 20,492 |
| 73 | prompt$:ab,ti | 104,313 |
| 74 | alert$:ab,ti | 46,870 |
| 75 | algorithm$:ab,ti | 337,237 |
| 76 | 'clinical audit'/de | 51,205 |
| 77 | (audit* NEAR/2 (clinical OR medical OR record$ OR chart$)):ab,ti | 11,160 |
| 78 | 'medical record review'/de | 135,164 |
| 79 | 'chart review*':ab,ti | 90,114 |
| 80 | 'feedback system'/de | 85,182 |
| 81 | 'negative feedback'/de | 17,511 |
| 82 | 'positive feedback'/de | 11,976 |
| 83 | feedback:ab,ti | 187,790 |
| 84 | 'benchmarking'/de | 6,011 |
| 85 | 'health personnel attitude'/de | 84,507 |
| 86 | 'physician attitude'/de | 54,305 |
| 87 | (attitude$ NEAR/2 (physician$ OR resident$ OR clinician$ OR provider$)):ab,ti | 7,540 |
| 88 | (belief$ NEAR/2 (physician$ OR resident$ OR clinician$ OR provider$)):ab,ti | 1,525 |
| 89 | behavio?ral AND change$:ab,ti | 39,094 |
| 90 | 'utilization review'/de | 64,305 |
| 91 | utili?ation:ab,ti | 291,793 |
| 92 | #44 OR #45 OR #46 OR #47 OR #48 OR #49 OR #50 OR #51 OR #52 OR #53 OR #54 OR #55 OR #56 OR #57 OR #58 OR #59 OR #60 OR #61 OR #62 OR #63 OR #64 OR #65 OR #66 OR #67 OR #68 OR #69 OR #70 OR #71 OR #72 OR #73 OR #74 OR #75 OR #76 OR #77 OR #78 OR #79 OR #80 OR #81 OR #82 OR #83 OR #84 OR #85 OR #86 OR #87 OR #88 OR #89 OR #90 OR #91 | 5,017,293 |
| 93 | 'unnecessary procedure'/de | 3,241 |
| 94 | (overuse:ab OR 'over use':ab) AND ,t | 3,327 |
| 95 | (decreas* NEAR/2 (referral* OR imaging OR radiograph* OR radiolog* OR ct OR 'computed tomography' OR 'x ray$' OR xray$ OR mri OR mris)):ab,ti | 5,102 |
| 96 | (reduc* NEAR/2 (referral* OR imaging OR radiograph* OR radiolog* OR ct OR 'computed tomography' OR 'x ray$' OR xray$ OR mri OR mris)):ab,ti | 10,950 |
| 97 | (increas* NEAR/2 (referral* OR imaging OR radiograph* OR radiolog* OR ct OR 'computed tomography' OR 'x ray$' OR xray$ OR mri OR mris)):ab,ti | 18,392 |
| 98 | (unnecessary NEAR/2 (referral* OR imaging OR radiograph* OR radiolog* OR ct OR 'computed tomography' OR 'x ray$' OR xray$ OR mri OR mris)):ab,ti | 2,083 |
| 99 | (appropriate* NEAR/2 (referral* OR imaging OR radiograph* OR radiolog* OR ct OR 'computed tomography' OR 'x ray$' OR xray$ OR mri OR mris)):ab,ti | 9,666 |
| 100 | (inappropriate* NEAR/2 (referral* OR imaging OR radiograph* OR radiolog* OR ct OR 'computed tomography' OR 'x ray$' OR xray$ OR mri OR mris)):ab,ti | 1,103 |
| 101 | #93 OR #94 OR #95 OR #96 OR #97 OR #98 OR #99 OR #100 | 51,322 |
| 102 | 'clinical trial'/exp | 1,575,203 |
| 103 | 'clinical trial (topic)'/exp | 344,153 |
| 104 | random*:ab,ti | 1,621,928 |
| 105 | 'health services research'/de | 34,173 |
| 106 | 'comparative study'/de | 908,209 |
| 107 | 'prospective study'/de | 659,529 |
| 108 | 'time series analysis'/de | 28,054 |
| 109 | prospective:ab,ti | 900,491 |
| 110 | (before NEAR/2 after):ab,ti | 400,592 |
| 111 | (pretest NEAR/1 posttest):ab,ti | 4,235 |
| 112 | pre AND post AND test*:ab,ti | 95,148 |
| 113 | pretesting:ab,ti | 2,051 |
| 114 | 'repeated measurement$':ab,ti | 12,305 |
| 115 | 'repeated measure$':ab,ti | 56,671 |
| 116 | 'time series':ab,ti | 37,453 |
| 117 | (control* NEAR/3 (study OR design OR trial OR matched OR group)):ab,ti | 1,254,252 |
| 118 | 'quasi-experimental':ab,ti | 17,752 |
| 119 | #102 OR #103 OR #104 OR #105 OR #106 OR #107 OR #108 OR #109 OR #110 OR #111 OR #112 OR #113 OR #114 OR #115 OR #116 OR #117 OR #118 | 5,397,090 |
| 120 | #22 AND #31 AND #43 AND (#92 OR #101) AND #119 | 1,416 |

**CINAHL Plus via EBSCOhost**

Search conducted 4 February 2021

| **#** | **Query** | **Results** |
| --- | --- | --- |
| S1 | (MH "Primary Health Care") | 65,496 |
| S2 | TI "primary healthcare" OR AB "primary healthcare" | 3,580 |
| S3 | TI (primary N2 care) OR AB (primary N2 care) | 86,463 |
| S4 | (MH "Community Medicine") | 410 |
| S5 | (MH "Community Health Centers") | 6,454 |
| S6 | (MH "Community Health Services") | 22,872 |
| S7 | (MH "Occupational Health Services") | 5,993 |
| S8 | TI community OR AB community | 253,011 |
| S9 | (MH "Ambulatory Care") | 12,833 |
| S10 | TI (ambulatory N2 (care OR setting#)) OR AB (ambulatory N2 (care OR setting#)) | 7,586 |
| S11 | (MH "Practitioner's Office") | 1,457 |
| S12 | TI clinic# OR AB clinic# | 119,831 |
| S13 | (MH "Physicians, Family") | 20,442 |
| S14 | (MH "Family Practice") | 25,461 |
| S15 | TI ( family N1 (medicine OR practice OR practitioner# OR physician# OR doctor#) ) OR AB ( family N1 (practice OR practitioner# OR physician# OR doctor#) ) | 13,144 |
| S16 | TI ( general N1 (practice or practioner#) ) OR AB ( general N1 (practice or practioner# ) ) | 17,694 |
| S17 | TI ( gp OR gps ) OR AB ( gp OR gps ) | 21,275 |
| S18 | (MH "Emergency Service") | 56,959 |
| S19 | (MH "Trauma Centers") | 6,635 |
| S20 | (MH "Emergency Medicine") | 12,740 |
| S21 | TI ( emergency N2 (department# OR unit# OR room# OR physician#) ) OR AB ( emergency N2 (department# OR unit# OR room# OR physician#) ) | 69,760 |
| S22 | TI ( trauma N2 (centre# OR center# OR department# OR unit#)) OR AB ( trauma N2 (centre# OR center# OR department# OR unit#)) | 9,975 |
| S23 | TI ( triage N2 (centre# OR center# OR department# OR unit#)) OR AB ( triage N2 (centre# OR center# OR department# OR unit#)) | 615 |
| S24 | TI ("accident and emergency" OR "accident & emergency") OR AB ("accident and emergency" OR "accident & emergency") | 2,618 |
| S25 | S1 OR S2 OR S3 OR S4 OR S5 OR S6 OR S7 OR S8 OR S9 OR S10 OR S11 OR S12 OR S13 OR S14 OR S15 OR S16 OR S17 OR S18 OR S19 OR S20 OR S21 OR S22 OR S23 OR S24 | 623,187 |
| S26 | (MH "Back Pain") | 11,368 |
| S27 | (MH "Low Back Pain") | 20,435 |
| S28 | TI ( back OR backache OR backpain ) OR AB ( back OR backache OR backpain ) | 74,381 |
| S29 | TI dorsalgia OR AB dorsalgia | 30 |
| S30 | (MH "Spine+") | 46,903 |
| S31 | (MH "Spinal Diseases+") | 35,440 |
| S32 | TI ( lumbar OR lumbosacral OR "lumbo sacral" OR spine OR spinal ) OR AB ( lumbar OR lumbosacral OR "lumbo sacral" OR spine OR spinal ) | 102,536 |
| S33 | S26 OR S27 OR S28 OR S29 OR S30 OR S31 OR S32 | 195,425 |
| S34 | (MH "Diagnostic Imaging") | 32,412 |
| S35 | MW "RA" | 116,621 |
| S36 | (MH "Radiography") | 16,401 |
| S37 | (MH "Magnetic Resonance Imaging") | 125,154 |
| S38 | (MH "Tomography, X-Ray Computed+") | 108,644 |
| S39 | TI imaging OR AB imaging | 169,130 |
| S40 | TI radiograph* OR AB radiograph* | 58,886 |
| S41 | TI radiolog* OR AB radiolog* | 61,784 |
| S42 | TI "computed tomography" OR AB "computed tomography" | 61,795 |
| S43 | TI ( "x ray#" OR xray# ) OR AB ( "x ray#" OR xray# ) | 25,028 |
| S44 | TI ( mri OR mris ) OR AB ( mri OR mris ) | 60,214 |
| S45 | S34 OR S35 OR S36 OR S37 OR S38 OR S39 OR S40 OR S41 OR S42 OR S43 OR S44 | 472,803 |
| S46 | (MH "Quality Improvement") | 57,627 |
| S47 | (MH "Program Evaluation") | 42,699 |
| S48 | TI ( program# OR programme# ) OR AB ( program# OR programme# ) | 364,678 |
| S49 | (MH "Education, Continuing") | 12,746 |
| S50 | (MH "Education, Medical, Continuing") | 7,869 |
| S51 | (MH "Staff Development") | 28,903 |
| S52 | MW "ED" | 202,204 |
| S53 | TI ( educat* OR teach* OR train OR training OR instruction* OR learn* ) OR AB ( educat* OR teach* OR train OR training OR instruction* OR learn* ) | 710,525 |
| S54 | TI ( lecture* OR seminar* OR presentation* OR tutorial* OR workshop* OR "work shop" OR "work shops" OR webinar* ) OR AB ( lecture* OR seminar* OR presentation* OR tutorial* OR workshop* OR "work shop" OR "work shops" OR webinar* ) | 129,044 |
| S55 | TI "academic detailing" OR AB "academic detailing" | 337 |
| S56 | (MH "Consumer Health Information") | 12,996 |
| S57 | TI "patient education" OR AB "patient education" | 11,868 |
| S58 | TI "consumer health" OR AB "consumer health" | 1,102 |
| S59 | (MH "Teaching Materials+") | 125,752 |
| S60 | (MH "Pamphlets") | 3,468 |
| S61 | TI ( leaflet# OR booklet# OR poster# OR pamphlet# ) OR AB ( leaflet# OR booklet# OR poster# OR pamphlet# ) | 20,115 |
| S62 | TI "knowledge translation" OR AB "knowledge translation" | 1,937 |
| S63 | (MH "Telecommunications") | 2,434 |
| S64 | (MH "Communications Media") | 10,939 |
| S65 | TI ( (written OR printed OR oral) N1 information ) OR AB ( (written OR printed OR oral) N1 information ) | 1,946 |
| S66 | TI ( (written or printed or oral) N1 communication# ) OR AB ( (written or printed or oral) N1 communication# ) | 1,034 |
| S67 | TI ( (media or communication?) N1 campaign# ) OR AB ( (media or communication?) N1 campaign# ) | 1,583 |
| S68 | TI "choosing wisely" OR AB "choosing wisely" | 718 |
| S69 | TI marketing OR AB marketing | 14,754 |
| S70 | (MH "Guideline Adherence") | 15,701 |
| S71 | TI ( guideline# N2 (adher* or comply* or complies or compliance or disseminat* or distribut*)) OR AB ( guideline# N2 (adher* or comply* or complies or compliance or disseminat* or distribut*)) | 6,000 |
| S72 | TI ( protocol# N2 (adher* or comply* or complies or compliance or disseminat* or distribut*) ) OR AB ( protocol# N2 (adher* or comply* or complies or compliance or disseminat* or distribut*) ) | 1,718 |
| S73 | TI ( policy N2 (change# or changing or modifi* or revise or revised or revision# or update#) ) OR AB ( policy N2 (change# or changing or modifi* or revise or revised or revision# or update#) ) | 7,985 |
| S74 | (MH "Decision Support Techniques") | 6,962 |
| S75 | (MH "Decision Support Systems, Clinical") | 5,448 |
| S76 | (MH "Reminder Systems") | 2,945 |
| S77 | TI "decision support" OR AB "decision support" | 6,764 |
| S78 | TI reminder# OR AB reminder# | 6,615 |
| S79 | TI prompt# OR AB prompt# | 18,731 |
| S80 | TI alert# OR AB alert# | 12,871 |
| S81 | TI algorithm# OR AB algorithm# | 36,243 |
| S82 | (MH "Audit") | 17,952 |
| S83 | (MH "Record Review") | 62,774 |
| S84 | TI ( audit* N2 (clinical OR medical OR record# OR chart#) ) OR AB ( audit* N2 (clinical OR medical OR record# OR chart#) ) | 4,153 |
| S85 | TI "chart review*" OR AB "chart review*" | 16,309 |
| S86 | (MH "Feedback") | 15,494 |
| S87 | TI feedback OR AB feedback | 35,968 |
| S88 | (MH "Benchmarking") | 7,475 |
| S89 | (MH "Attitude of Health Personnel") OR (MH "Physician Attitudes") | 61,271 |
| S90 | TI ( attitude# N2 (physician# OR resident# OR clinician# OR provider#) ) OR AB ( attitude# N2 (physician# OR resident# OR clinician# OR provider#) ) | 3,583 |
| S91 | TI ( belief# N2 (physician# OR resident# OR clinician# OR provider#) ) OR AB ( belief# N2 (physician# OR resident# OR clinician# OR provider#) ) | 945 |
| S92 | TI "behavio?ral change#" OR AB "behavio?ral change#" | 1,602 |
| S93 | (MH "Practice Patterns") | 12,713 |
| S94 | (MH "Utilization Review") | 2,176 |
| S95 | TI utili?ation OR AB utili?ation | 57,393 |
| S96 | S46 OR S47 OR S48 OR S49 OR S50 OR S51 OR S52 OR S53 OR S54 OR S55 OR S56 OR S57 OR S58 OR S59 OR S60 OR S61 OR S62 OR S63 OR S64 OR S65 OR S66 OR S67 OR S68 OR S69 OR S70 OR S71 OR S72 OR S73 OR S74 OR S75 OR S76 OR S77 OR S78 OR S79 OR S80 OR S81 OR S82 OR S83 OR S84 OR S85 OR S86 OR S87 OR S88 OR S89 OR S90 OR S91 OR S92 OR S93 OR S94 OR S95 | 1,544,451 |
| S97 | (MH "Unnecessary Procedures") | 3,631 |
| S98 | TI ( overuse OR "over use" ) OR AB ( overuse OR "over use" ) | 6,043 |
| S99 | TI ( decreas* N2 (referral* OR imaging OR radiograph* OR radiolog* OR "CT scan*" OR "computed tomography" OR "x ray#" OR xray# OR mri OR mris) ) OR AB ( decreas* N2 (referral* OR imaging OR radiograph* OR radiolog* OR "CT scan*" OR "computed tomography" OR "x ray#" OR xray# OR mri OR mris) ) | 1,035 |
| S100 | TI ( reduc* N2 (referral* OR imaging OR radiograph* OR radiolog* OR "CT scan*" OR "computed tomography" OR "x ray#" OR xray# OR mri OR mris) ) OR AB ( reduc* N2 (referral* OR imaging OR radiograph* OR radiolog* OR "CT scan*" OR "computed tomography" OR "x ray#" OR xray# OR mri OR mris) ) | 2,880 |
| S101 | TI ( increas* N2 (referral* OR imaging OR radiograph* OR radiolog* OR "CT scan*" OR "computed tomography" OR "x ray#" OR xray# OR mri OR mris) ) OR AB ( increas* N2 (referral* OR imaging OR radiograph* OR radiolog* OR "CT scan*" OR "computed tomography" OR "x ray#" OR xray# OR mri OR mris) ) | 4,670 |
| S102 | TI ( unnecessary N2 (referral* OR imaging OR radiograph* OR radiolog* OR "CT scan*" OR "computed tomography" OR "x ray#" OR xray# OR mri OR mris) ) OR AB ( unnecessary N2 (referral* OR imaging OR radiograph* OR radiolog* OR "CT scan*" OR "computed tomography" OR "x ray#" OR xray# OR mri OR mris) ) | 670 |
| S103 | TI ( appropriate* N2 (referral* OR imaging OR radiograph* OR radiolog* OR "CT scan*" OR "computed tomography" OR "x ray#" OR xray# OR mri OR mris) ) OR AB ( appropriate* N2 (referral* OR imaging OR radiograph* OR radiolog* OR "CT scan*" OR "computed tomography" OR "x ray#" OR xray# OR mri OR mris) ) | 3,591 |
| S104 | TI ( inappropriate* N2 (referral* OR imaging OR radiograph* OR radiolog* OR "CT scan*" OR "computed tomography" OR "x ray#" OR xray# OR mri OR mris) ) OR AB ( inappropriate* N2 (referral* OR imaging OR radiograph* OR radiolog* OR "CT scan*" OR "computed tomography" OR "x ray#" OR xray# OR mri OR mris) ) | 346 |
| S105 | S97 OR S98 OR S99 OR S100 OR S101 OR S102 OR S103 OR S104 | 21,655 |
| S106 | (MH "Clinical Trials+") | 313,763 |
| S107 | TI random* OR AB random* | 366,329 |
| S108 | (MH "Health Services Research") | 15,574 |
| S109 | (MH "Comparative Studies") | 363,421 |
| S110 | (MH "Controlled Before-After Studies") | 194 |
| S111 | (MH "Prospective Studies") | 460,876 |
| S112 | (MH "Quasi-Experimental Studies+") | 16,223 |
| S113 | TI prospective OR AB prospective | 185,528 |
| S114 | TI before N2 after OR AB before N2 after | 73,876 |
| S115 | TI pretest N1 posttest OR AB pretest N1 posttest | 4,785 |
| S116 | TI "pre post test*" OR AB "pre post test*" | 765 |
| S117 | TI pretesting OR AB pretesting | 440 |
| S118 | TI "repeated measurement#" OR AB "repeated measurement#" | 2,238 |
| S119 | TI "repeated measure#" OR AB "repeated measure#" | 18,670 |
| S120 | TI "time series" OR AB "time series" | 6,164 |
| S121 | TI ( control* N3 (study OR design OR trial OR matched OR group) ) OR AB ( control* N3 (study OR design OR trial OR matched OR group) ) | 318,068 |
| S122 | TI "quasi experimental" OR AB "quasi experimental" | 11,005 |
| S123 | S106 OR S107 OR S108 OR S109 OR S110 OR S111 OR S112 OR S113 OR S114 OR S115 OR S116 OR S117 OR S118 OR S119 OR S120 OR S121 OR S122 | 1,329,969 |
| S124 | S25 AND S33 AND S45 AND (S96 OR S105) AND S123 | 425 |

**CENTRAL via Cochrane Library**

Search conducted 4 February 2021

| **ID** | **Search** | **Hits** |
| --- | --- | --- |
| #1 | ("primary care" OR "primary health care" OR "primary healthcare"):ti,ab,kw | 21001 |
| #2 | (community):ti,ab,kw | 44056 |
| #3 | ("ambulatory care" OR "ambulatory setting" OR "ambulatory settings"):ti,ab,kw | 5055 |
| #4 | (clinic OR clinics):ti,ab,kw | 53723 |
| #5 | ("family medicine" OR "family practice" OR "family physician" OR "family physicians" OR "family practitioner" OR "family practitioners" OR "family doctor" OR "family doctors"):ti,ab,kw | 4034 |
| #6 | ("general practice" OR "general practitioner" OR "general practitioners"):ti,ab,kw | 10956 |
| #7 | ("emergency department" OR "emergency departments" OR "emergency room" OR "emergency rooms" OR "emergency medicine"):ti,ab,kw | 13928 |
| #8 | ("trauma centre" OR "trauma centres" OR "trauma center" OR "trauma centers" OR "trauma unit" OR "trauma units"):ti,ab,kw | 1183 |
| #9 | ("triage department" OR "triage departments" OR "triage unit" OR "triage units"):ti,ab,kw | 1 |
| #10 | ("accident and emergency" OR "accident & emergency"):ti,ab,kw | 333 |
| #11 | {OR #1-#10} | 129104 |
| #12 | (back OR backache OR backpain):ti,ab,kw | 28792 |
| #13 | (dorsalgia):ti,ab,kw | 106 |
| #14 | (lumbar OR lumbosacral OR "lumbo sacral" OR spine OR spinal):ti,ab,kw | 43722 |
| #15 | {OR #12-#14} | 65146 |
| #16 | (imaging):ti,ab,kw | 68538 |
| #17 | (radiograph*):ti,ab,kw | 23618 |
| #18 | (radiolog*):ti,ab,kw | 15120 |
| #19 | ("ct scan" OR "ct scans"):ti,ab,kw | 6108 |
| #20 | ("computed tomography"):ti,ab,kw | 12632 |
| #21 | (("x ray" OR "x rays" OR xray OR xrays)):ti,ab,kw | 19708 |
| #22 | (mri OR mris):ti,ab,kw | 24392 |
| #23 | {OR #16-#22} | 116095 |
| #24 | (program OR programs OR programme OR programmes):ti,ab,kw | 120775 |
| #25 | (educat* OR teach* OR train OR training OR instruction* OR learn*):ti,ab,kw | 192700 |
| #26 | (lecture* OR seminar* OR presentation* OR tutorial* OR workshop* OR "work shop" OR "work shops" OR webinar*):ti,ab,kw | 24501 |
| #27 | ("academic detailing"):ti,ab,kw | 303 |
| #28 | ("patient education"):ti,ab,kw | 13664 |
| #29 | ("consumer health"):ti,ab,kw | 241 |
| #30 | (leaflet OR leaflets OR booklet OR booklets OR poster OR posters OR pamphlet OR pamphlets):ti,ab,kw | 8392 |
| #31 | ("knowledge translation"):ti,ab,kw | 321 |
| #32 | ("written information" OR "printed information" OR "oral information"):ti,ab,kw | 1418 |
| #33 | ("written communications" OR "printed communications" OR "oral communications"):ti,ab,kw | 6 |
| #34 | ("media campaign" OR "media campaigns" OR "communication campaign" OR "communication campaigns" OR "communications campaign" OR "communications campaigns"):ti,ab,kw | 244 |
| #35 | ("choosing wisely"):ti,ab,kw | 55 |
| #36 | (marketing):ti,ab,kw | 2312 |
| #37 | ("guideline adherence" OR "guideline compliance" OR "guideline dissemination" OR "guideline distribution"):ti,ab,kw | 1338 |
| #38 | ("protocol adherence" OR "protocol complaince"):ti,ab,kw | 416 |
| #39 | ("policy change" OR "policy changes" OR "policy modifications" OR "policy revisions" OR "policy updates"):ti,ab,kw | 217 |
| #40 | ("decision support"):ti,ab,kw | 3042 |
| #41 | (reminder OR reminders):ti,ab,kw | 5614 |
| #42 | (prompt OR prompts):ti,ab,kw | 4250 |
| #43 | (alert OR alerts):ti,ab,kw | 2731 |
| #44 | (algorithm OR algorithms):ti,ab,kw | 13242 |
| #45 | ("clinical audit" OR "medical audit" OR "record audit" OR "chart audit" OR "chart review"):ti,ab,kw | 2704 |
| #46 | (feedback):ti,ab,kw | 17687 |
| #47 | ("physician attitudes" OR "resident attitudes" OR "clinician attitudes" OR "provider attitudes"):ti,ab,kw | 146 |
| #48 | ("physician beliefs" OR "resident beliefs" OR "clinician beliefs" OR "provider beliefs"):ti,ab,kw | 18 |
| #49 | ("behavioral change" OR "behavioral changes" OR "behavioural change" OR "behavioural changes"):ti,ab,kw | 2482 |
| #50 | (utilization OR utilisation):ti,ab,kw | 17855 |
| #51 | {OR #24-#50} | 309229 |
| #52 | (overuse OR "over use"):ti,ab,kw | 1137 |
| #53 | (decreas* NEAR/2 (referral* OR imaging OR radiograph* OR radiolog* OR CT OR "computed tomography" OR "x ray" OR "x rays" OR xray OR xrays OR mri OR mris)):ti,ab,kw | 337 |
| #54 | (reduc* NEAR/2 (referral* OR imaging OR radiograph* OR radiolog* OR CT OR "computed tomography" OR "x ray" OR "x rays" OR xray OR xrays OR mri OR mris)):ti,ab,kw | 1349 |
| #55 | (increas* NEAR/2 (referral* OR imaging OR radiograph* OR radiolog* OR CT OR "computed tomography" OR "x ray" OR "x rays" OR xray OR xrays OR mri OR mris)):ti,ab,kw | 892 |
| #56 | (unnecessary NEAR/2 (referral* OR imaging OR radiograph* OR radiolog* OR CT OR "computed tomography" OR "x ray" OR "x rays" OR xray OR xrays OR mri OR mris)):ti,ab,kw | 74 |
| #57 | (appropriate* NEAR/2 (referral* OR imaging OR radiograph* OR radiolog* OR CT OR "computed tomography" OR "x ray" OR "x rays" OR xray OR xrays OR mri OR mris)):ti,ab,kw | 349 |
| #58 | (inappropriate* NEAR/2 (referral* OR imaging OR radiograph* OR radiolog* OR CT OR "computed tomography" OR "x ray" OR "x rays" OR xray OR xrays OR mri OR mris)):ti,ab,kw | 46 |
| #59 | {OR #52-#58} | 4029 |
| #60 | #11 AND #15 AND #23 AND (#51 OR #59) | 406 |
| #61 | #11 AND #15 AND #23 AND (#51 OR #59) in Trials | 380 |
