# Supplementary material for: What behaviour change techniques have been used to improve adherence to evidence-based low back pain imaging?
Source: Implement Sci. 2021 Jul 2;16:68. doi: 10.1186/s13012-021-01136-w (PMC8254222; doi:10.1186/s13012-021-01136-w)
Supplement: Supplementary file 2 — Additional file 2. [file 13012_2021_1136_MOESM2_ESM.docx]

**Coding Rules (based on feedback from team at BCT meeting May 28^th^, 2020)**

**Patient education materials**

If patient education materials are delivered to physician to use with patient, then code as

- Pamphlet/booklet/leaflet – 12.5 add object to environment
- Prescription pad – 12.5 add object to environment
- Poster – 7.1 prompt/cue

If the research team indicates that the patient education material (booklet or prescription pad) was intended to act as a reminder then also code as 7.1 Prompt cue, otherwise only code as 12.5.

If Patient education material delivered directly to patients from researchers (or someone other than the physician) – do not code.

**Patient as mechanism of action**

If the patient is given something (that relates to the behaviour in question, e.g. a letter describing the risks/harms of imaging and that they are not useful in diagnosing most back pain) that they need to take with them to their appointment to discuss with the physician – note these and we will discuss as a team – could possibly code as “restructuring social environment”, but unclear right now.

**Removing or restricting ordering**(see Fine, Ip, Graves. Tracey)

- Hard stop (unable to order) – 12.1 Restructuring the physical environment
- Order set is modified (or new info required on order form, etc.) – 12.1 Restructuring the physical environment
- Soft stop (Order is sent back) and need to provide more info before continuing – 12.1 Restructuring the physical environment
- Soft stop (Order is sent back) **and** have to talk to somebody before order can proceed – 12.1 and 12.2 restructuring social environment

The type of information provided to physicians in the Jarvik study

“The consequence of performing this behaviour (imaging) is finding nothing helpful on the image for diagnosis or treatment”

Code as both:

·        5.1 information about health consequences

·        2.7 feedback on outcomes of behaviour

**When there are guidelines used as part of the study intervention**

- If the guideline producer is listed and is known to be reputable (e.g. NICE) – code 9.1 credible source
- If the guideline producer is a “society” – review with team
- If there is no reference to the guideline producer provided – no dot code

When Information is provided in a “workshop”

- Code 4.1
- Only code at 6.1 and 8.1 if there are explicitly states examples of this. This is because we can’t assume a “workshop” will include demonstration or practice it could just be didactic, workshop is a vague term, dissimilar from “cookery class or exercise group” in which it is more clear that demonstration and practice is inherent.

**Inclusion of case studies/patient vignettes**

- Coded as 4.1 unless there was explicit evidence of 6.1 or 8.1.

**Academic detailing**

- Code academic detailing as 3.1. social support (unspecified). Often the precise content was not specified. We agreed to treat academic detailing like other ‘packages’ in the BCT taxonomy v1 that are not well defined and code it as ‘social support (unspecified)’. This is based on the idea that the health professional is getting some unspecified support, but we cannot precisely pin it down. If the paper included a detailed description of the content of the academic detailing we added other BCT codes, if indicated.

**Online decision support systems**

- If a decision support system was added to the ordering process that provided the GP with information about when to order / not order an image or if their order was considered adherent or not at the time of referral as a means of reminding them about the guidelines to assist them with their referral, this was coded as both 12.5 and 7.1

**Continuing education credits**

- We did not code “awarded Education credits” unless the education credit was contingent on performing the behavior and not just attending the education session.
